# Adiponectin Protects Against Cerebral Ischemic Injury Through AdipoR1/AMPK Pathways

**DOI:** 10.3389/fphar.2019.00597

**Published:** 2019-05-22

**Authors:** Bin Liu, Jing Liu, Jiangong Wang, Fengjiao Sun, Shujun Jiang, Fengai Hu, Dan Wang, Dunjiang Liu, Cuilan Liu, Haijing Yan

**Affiliations:** Institute for Metabolic and Neuropsychiatric Disorders, Binzhou Medical University Hospital, Binzhou, China

**Keywords:** adiponectin, AdipoRon, ischemia, adiponectin receptor 1, amp-activated protein kinase, PGC-1α, mitochondrial

## Abstract

Excitotoxicity induced by excessive N-methyl-D-aspartate (NMDA) receptor activation underlies the pathology of ischemic injury. Adiponectin (APN) is an adipocyte-derived protein hormone that modulates a number of metabolic processes. APN exerts a wide range of biological functions in the central nervous system. However, the role of APN and its receptors in cerebral ischemia/reperfusion (I/R)-induced injury and the related mechanisms remain to be clarified. Here, we found that APN and APN receptor agonist AdipoRon (APR) were protective against excitotoxicity induced by oxygen and glucose deprivation/reperfusion (OGD/R) and NMDA in primary neurons. Adiponectin receptor 1 (AdipoR1) knockdown reversed the protection conferred by either APN or APR. Moreover, the protective effects offered by both APN and APR were compromised by compound C, an inhibitor of amp-activated protein kinase (AMPK) phosphorylation. Both APN and APR protected the dissipation of the ΔΨm caused by OGD/R. They also up-regulated the PGC-1α expression, which was reversed by compound C. Furthermore, both APN and APR ameliorated but APN knockout aggravated the infarct volume and neurological deficient induced by transient middle cerebral artery occlusion (tMCAO) *in vivo*. Taken together, these findings show that APN and APR protect against ischemic injury *in vitro* and *in vivo*. The protective mechanism is mainly related to AdipoR1-dependent AMPK phosphorylation and PGC-1α up-regulation.

## Introduction

Under physiological condition, glutamate mainly involves in synaptic plasticity, learning, and memory physiological process; however in pathological condition, it tends to show neuronal excitotoxicity (Wu and Tymianski, 2018), which is caused by overactivated NMDA receptor by excessive extracellular glutamate (Rothman and Olney, 1995). Abnormal function of the glutamatergic system has been implicated in the pathophysiology of stroke (Dirnagl et al., 1999).

Adiponectin (APN) is a fat-derived secreted protein negatively associated with fat accumulation (Hu et al., 1996; Diez and Iglesias, 2003). APN is able to cross the blood–brain barrier (BBB) (Kubota et al., 2007; Kusminski et al., 2007), and binds with its receptors (AdipoR1 and AdipoR2) to activate numerous signaling pathways, including amp-activated protein kinase (AMPK) and p38 mitogen-activated protein kinase (p38 MAPK), to regulate cell functions (Scherer et al., 1995; Mao et al., 2006; Thundyil et al., 2012). AdipoR1 is widely expressed in the brain, whereas AdipoR2 is barely expressed in the brain (Hug et al., 2004). In the central nervous system (CNS), AdipoR1 is mainly expressed in neurons and is involved in the regulation of energy metabolism (Thundyil et al., 2012).

APN was primarily regarded important in regulating the functions, such as body weight, endothelial function, insulin-sensitization, anti-atherogenic, and anti-inflammatory actions (Ouchi et al., 1999; Yamauchi et al., 2001; Diez and Iglesias, 2003). Recently, many studies indicate that APN plays an important role in the regulation of CNS pathologies, such as depression, AD, epilepsy, and stroke. The APN peripheral levels were significantly lower in major depressive disorder patients (Carvalho et al., 2014), and administration of APN elicited antidepressant-like behavioral effects in mice through the inhibition of GSK-3β (Liu et al., 2012). The serum APN also significantly decreased in AD patients (Teixeira et al., 2013), and APN was protective against amyloid β-induced neurotoxicity in Alzheimer’s disease through AMPK activation and NF-κB suppression (Chan et al., 2012). Decreased serum APN and increased hippocampal AdipoR1 were found in the hippocampus of KA-treated seizure mice (Jeon et al., 2009). APN was neuroprotective against seizures through preserving the integrity of the BBB (Jeon et al., 2009). However, APN deficiency exacerbated seizure-related brain injury (Lee et al., 2011). Thus, APN plays an important role in neurological disorders.

APN level is significantly reduced, and APN reversed the decreased cerebral blood flow (CBF) during cerebral ischemia (Nishimura et al., 2008). APN protects against glutamate-induced excitotoxicity in HT22 hippocampal neurons (Yue et al., 2016;Wang et al., 2018). APN also protects against ischemia in diabetic mice (Song et al., 2015). However, the protective mechanism of APN on cerebral ischemic injury remains to be studied in the neurons of cortex, which is the main damage area of ischemia. In this article, we will study the effects of APN and APN receptor agonist AdipoRon (APR) on OGD/R-induced excitotoxicity the further investigate the related mechanisms in primary neurons.

## Materials and Methods

### Cell Culture

For primary neuronal cell culture, pregnant C57BL6J mice were anesthetized by intraperitoneal injection of chloral hydrate, and the cortex was isolated from embryos (18 days). Cells (800–1,000 cells/mm^2^) were seeded on coverslips coated with 30 mg/ml poly-d-lysine. Cells were placed in fresh serum-free neurobasal medium (21103, Gibco) plus 2% B27 and fed every 4 days with fresh medium and used after 7 days (DIV7).

### Oxygen-Glucose Deprivation and Drug Administration

Cells were rinsed twice with warm glucose-free dulbecco’s modified eagle medium (DMEM) (Gibco), and refreshed with O_2_- and glucose-free DMEM (pre-balanced in an O_2_-free chamber at 37°C). Cells were then immediately placed in a sealed chamber (Billups Rothenburg, MIC-101) loaded with mixed gas containing 5% CO_2_ and 95% N_2_ for 5 min at 25 L/min. The primary neurons were then incubated at 37°C for 2 h before reperfusion. For reperfusion, cells were refreshed with normal culture medium. The indicated concentrations of APN was dissolved in PBS, whereas APR and compound C were dissolved in dimethyl sulfoxide. APN and APR were administrated 0.5 h, whereas compound C was administrated 1 h before OGD treatment. Control cells were given equal refreshment but were incubated in glucose-containing DMEM for 2 h for OGD/R. The indicated concentrations of the chemicals were administrated on the basis of previous studies (Zhang et al., 2011; Domise et al., 2016; Zhang et al., 2017).

### MTT Assay

Cell viability was determined by 3-(4,5-dimethylthiazol-2-yl)-2,5-diphenyltetrazolium bromide (MTT) assay. Briefly, neurons were incubated with 0.5 mg/ml MTT for 2 h at 37°C after OGD/R treatment. The supernatant layer was removed, and 100 µl of dimethyl sulfoxide was added into each well. MTT metabolism was quantitated spectrophotometrically at 570 nm in a Biorad microplate reader. Results were expressed as the percentage of MTT reduction, taking the absorbance of control cells as 100%.

### Apoptosis Determined by TUNEL Assay

Apoptotic cells were determined by terminal deoxynucleotidyl transferase dUTP nick-end labeling (TUNEL) assay (Roche), and the total cell number was counted after DAPI staining. Five random fields were observed on each coverslip, and the experiments were repeated independently three times. The results were expressed as the percentage of TUNEL^+^/DAPI^+^ cells in the sections.

### Immunohistochemistry

Immunostaining was also performed in cultured neurons. Neurons seeded on coverslips were fixed in cold methanol for 10 min, and then incubated in 5% Bovine serum albumin (BSA) for 2 h to block nonspecific binding of IgG. Then the cells were reacted with antibody at 4°C overnight. The primary antibodies used in this experiment were MAP2 (MAB2418, Millipore, 1:300). After repeated washes in phosphate-buffered saline (PBS), cells were incubated with secondary antibody in 3% BSA for 2 h at 25°C. The secondary antibodies used in this experiment were goat anti-mouse IgG-AlexaFluo 594 (1:300, A11005; Invitrogen). After further washing in PBS, cultures were dried, coverslipped, and mounted on glass slides. The stained cells were observed under a fluorescence microscope (Olympus BX51, Japan). Total dendritic length and neuronal complexity were quantified by using ImageJ software and the Fiji plugins Simple Neurite Tracer Analysis as well as Sholl Analysis.

### Western Blot

Cultured neurons were lysed in ice-cold lysis buffer containing (in mmol/L): 50 Tris-HCl, 150 NaCl, 1% NP-40, 2 EDTA, 1 Na_3_VO_4_, pH 7.4) after 24 h of reperfusion. Mice were anesthetized by intraperitoneal injection of chloral hydrate (400 mg/kg), sacrificed 24 h after tMCAO or sham operation, and the brain was quickly removed and was immediately put in −40°C for 5 min. The separated tissue was lysed in ice-cold lysis buffer. After clearing debris by centrifugation at 14,000×*g* at 4°C, the protein concentration in the extracts was determined by the Bradford assay (Thermo, Hercules, CA). The precipitates were denatured with SDS sample loading buffer and separated on 10% SDS-PAGE. Proteins were transferred onto nitrocellulose membranes using a Bio-Rad mini-protein-III wet transfer unit overnight at 4°C. Transfer membranes were then incubated with blocking solution (5% nonfat dried milk dissolved in tris buffered saline tween (TBST) buffer (in mM): 10 Tris-HCl, 150 NaCl, and 0.1% Tween-20) for 1 h at room temperature, washed three times, and incubated with primary antibody for 2 h at room temperature. The primary antibodies used in this experiment were AdipoR1 (ab70362; Abcam, 1:1,000), AdipoR2 (ab77612, Abcam; 1:1,000), β-Actin (4970, Cell Signaling Technology, 1:1,000), Phospho-AMPK (2535, Cell Signaling Technology, 1:1,000), AMPK (2532, Cell Signaling Technology, 1:1,000), PGC-1α (ab54481; Abcam, 1:1,000), GAPDH (1:3,000; KC-5G4, KangChen Bio-tech, Shanghai). Membranes were washed three times in TBST buffer and incubated with the appropriate secondary antibodies (Odyssey, LI-COR, 1:5,000 dilution) for 2 h. Images were acquired with the Odyssey infrared imaging system and analyzed as specified in the Odyssey software manual. The results were expressed as the target protein/GAPDH or β-actin ratio and then normalized to the values measured in the control groups (presented as 100%).

### RNA Interference

Small-interfering RNA (siRNA) targeting mouse AdipoR1 were synthesized by corporation (GenePharm, Shanghai) as follows: negative control (sense: 5′-UUCUCCGAACGUGUCACGUTT-3′, antisense: 3′-ACGUGACACGUUCGGAGAATT-5′); sequence 1: (sense: 5′-AGGAGUUCGUGUAUAAGGUTT-3′, antisense: 5′-ACCU UAUACACGAACUCCUTT-3′); sequence 2: (sense: 5′-ACCAAAUAUGUACUU CAUGTT-3′, antisense: 5′-CAUGAAGUACAUAUUUGGUTT-3′); sequence 3: (sense: 5′-GGCUCUAUUACUCCUUCUATT-3′, antisense: 5′-UA GAAGGAGUAAUAGAGCCTT-3′). Primary neurons were transfected on DIV5, with 20 nmol AdipoR1 or negative control siRNA using Lipofectamine RNAiMAX (Invitrogen). After transfection in antibiotic-free medium for 8 h, cells were refreshed with normal medium. Experiments were performed 72 h after transfection.

### Mitochondrial Membrane Potential Assessment

The changes in relative mitochondrial membrane potential (ΔΨm) were assessed by using the lipophilic cationic probe 5,5′,6,6′-tetrachloro-1,1′,3,3′-tetraethylbenzamid azolocarbocyanine iodide (JC-1; Molecular Probes). The dye JC-1 undergoes a reversible change in fluorescence emission from green to greenish orange as ΔΨm increases. Cells with high ΔΨm form JC-1 aggregates and fluoresce red; those with low ΔΨm contain monomeric JC-1 and fluoresce green. After 2-h OGD and 24-h reperfusion, culture medium was removed and the cells, grown on coverslips, were incubated in the dark with JC-1 at a final concentration of 1.5 μM for 20 min. The cells were rinsed with PBS and excited at 488 nm with an Olympus BX-51 fluorescence microscope.

### Animals

Adult male WT and APN-KO mice (all C57BL/6 strain) were purchased from Shanghai Biomodel Organism Science & Technology Development Co. Ltd (Shanghai, China). Male WT mice and APN-KO mice weighing 22 to 25 g were used. For primary cortical neuronal culture, pregnant mice with embryonic (E18) fetuses were used. Mice were housed in separate cages under standard conditions, with a 12 h light/dark cycle (lights on at 9:00 am), and with *ad libitum* access to food and water. All experiments and protocols were approved by and conducted in accordance with the ethical guidelines of the Bin Zhou Medical University Animal Experimentation Committee and were in complete compliance with the National Institutes of Health Guide for the Care and Use of Laboratory Animals. Efforts were made to minimize any pain or discomfort, and the minimum number of animals was used.

### Transient MCAO Models and Drug Treatment

Mice were fasted overnight and anesthetized by intraperitoneal injection of chloral hydrate (400 mg/kg). Transient focal cerebral ischemia was induced by transient middle cerebral artery occlusion (tMCAO) (Yan et al., 2014). Briefly, a 6-0 nylon monofilament suture, blunted at the tip and coated with 1% poly-l-lysine, was advanced ∼10 mm into the internal carotid to occlude the origin of the middle cerebral artery (MCA). Reperfusion was allowed after 1 h by monofilament removal. Body temperature was maintained at 37°C with a heat lamp (FHC; Bowdoinham, ME, USA) during surgery and for 2 h after the start of reperfusion. CBF was determined in the territory of the MCA by laser Doppler flowmetry (Moor Instruments Ltd). A flexible fiber-optic probe was affixed to the skull over the cortex supplied by the proximal part of the right MCA (2 mm caudal to bregma and 6 mm lateral to midline). Animals with <80% reduction in CBF in the core of the MCA territory were excluded from the study. WT mice were randomly divided into separate four groups of four to five mice. Mice were given an intracerebroventricular injection of APN (1 µl of 0.3 mg/ml dissolved in saline) and APR (1 µl of 1 mg/ml dissolved in dimethyl sulfoxide) at 0.5 h before tMCAO, whereas compound C (1 µl of 40 mg/ml dissolved in dimethyl sulfoxide) at 1 h before tMCAO. Mice in control group were given equal saline or dimethyl sulfoxide. The indicated concentrations of the chemicals were administrated on the basis of previous studies (Wen et al., 2012; Zhang et al., 2016; Zhang et al., 2017).

Neurologic deficit scores were evaluated at 24 h of reperfusion as follows: 0, no deficit; 1, flexion of the contralateral forelimb on lifting of the whole animal by the tail; 2, circling to the contralateral side; 3, falling to the contralateral side; and 4, no spontaneous motor activity (Longa et al., 1989).

The infarct volume was determined at 24 h of reperfusion. The brains were quickly removed, sectioned coronally at 2-mm intervals and stained by immersion in the vital dye 2,3,5-triphenyltetrazolium hydrochloride (0.25%) at 37°C for 30 min. The extents of the normal and infarcted areas were analyzed using ImageJ (National Institutes of Health, Bethesda, MD, USA) and determined by the indirect method, which corrects for edema (contralateral hemisphere volume minus non-ischemic ipsilateral hemisphere volume). The percentage of the corrected infarct volume was calculated by dividing the infarct volume by the total contralateral hemispheric volume, and this ratio was then multiplied by 100.

### Statistical Analyses

Results are expressed as mean ± SEM. Statistical analysis was performed by one-way ANOVAs, followed by Tukey’s *post hoc* comparisons, using Prism software. *P* value <0.05 was considered statistically significant.

## Results

### Effects of APN and APR on Neuronal Viability on OGD/R- and NMDA-Induced Injury

**Figure 1:**
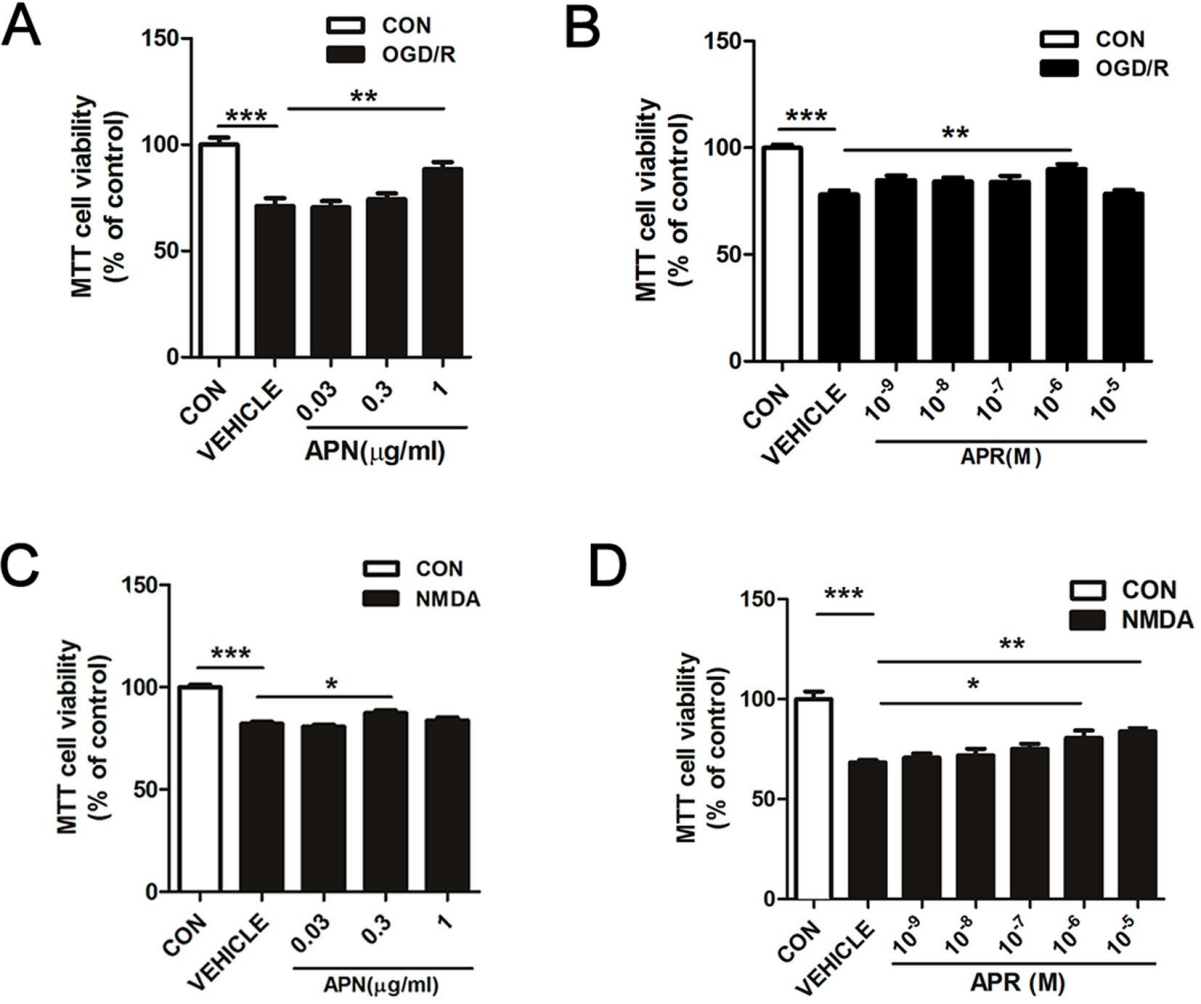
Effects of APN and APR on OGD/R-induced and NMDA-induced cell viability in promary neurons.** (A)** When APN (0.5 h before OGD treatment) was administered, cell viability was tested in primary neurons by MTT assay after OGD/R-induced injury (n = 6 per condition; **P <0.01, ***P <0.001 with ANOVAs followed by Tukey’s *post hoc* test). Data are presented as mean ± SEM. **(B)** When APR (0.5 h before OGD treatment) was administered, cell viability was tested in primary neurons by MTT assay after OGD/R-induced injury (n = 8-14 per condition; **P <0.01, ***P <0.001 with ANOVAs followed by Tukey’s *post hoc* test). Data are presented as mean ± SEM. **(C)** When APN (0.5 h before NMDA treatment) was administered, cell viability was tested in primary neurons by MTT assay after NMDA-induced (200 μM, 2 h) (n = 9-14 per condition; *P < 0.05, ***P < 0.001 with ANOVAs followed by Tukey’s *post hoc* test). Data are presented as mean ± SEM. **(D)** When APR (0.5 h before NMDA treatment) was administered, cell viability was tested in primary neurons by MTT assay after NMDA-induced (200 μM, 2 h) injury (n = 7 per condition; *P <0.05, **P <0.01, ***P <0.001 with ANOVAs followed by Tukey’s *post hoc* test). Data are presented as mean ± SEM.

Studies have shown that APN levels are significantly down-regulated in the brains of ischemic stroke victims (Pera et al., 2013), suggesting that low levels of APN might increase the risk of stroke. To figure out whether APN is protective against ischemic injury, two *in vitro* ischemic models on primary neurons were used. To investigate the role of APN on OGD/R-induced injury in primary neurons, various doses of APN on cell viability were detected by MTT. The results indicated that under OGD/R, the cell viability declined to 71.00 ± 3.32% of control (*P* <0.001, **Figure 1A**). APN could rescue neurons from OGD/R-induced cell viability impairment, as cell viability increased significantly to 88.47 ± 3.38% (*P* <0.01, **Figure 1A**) when APN (1 µg/ml) was administrated. However, no significant difference in cell viability was observed between vehicle (0 µg/ml) and low dose of APN (0.03, 0.3 µg/ml) groups (*P* > 0.05). Therefore, a dose of 1 µg/ml APN is selected for the later study. APR is an agonist of APN receptors, and then we also assessed the role of APR in OGD/R-induced injury. The results showed that the cell viability increased significantly from 78.11 ± 1.81% to 89.96 ± 2.34% (*P* <0.01, **Figure 1B**) when APR (10^-6^ M) was administrated, suggesting the APR was also protective against OGD/R-induced injury. Above all, these results told us that APN and APR protected neurons against OGD/R-induced injury in primary neurons.

Studies have demonstrated that excitotoxicity plays an important role in the pathological processes of ischemic brain injury (Rothman and Olney, 1995). To study the effects of APN and its receptors on neuronal excitotoxicity, an NMDA-induced excitotoxic model on primary neurons were used. The results showed that primary neurons exposed to NMDA exhibited a decreased cell viability of 80.20 ± 0.74% (*P* <0.001, **Figure 1C**), and APN (0.3 µg/ml) rescued the viability to 87.35 ± 1.29% (*P* <0.05, **Figure 1C**). Moreover, APR (10^-6^ M) rescued the viability from 78.34 ± 1.93% to 95.98 ± 3.58% (*P* <0.05, **Figure 1D**), and APR (10^-5^ M) rescued the viability to 98.16 ± 2.76% (*P* <0.01, **Figure 1D**). Above all, these results suggested that APN and APR were protective against NMDA-induced excitotoxicity in primary neurons.

### Effects of APN and APR on Apoptosis and Neuronal Morphology on OGD/R

**Figure 2:**
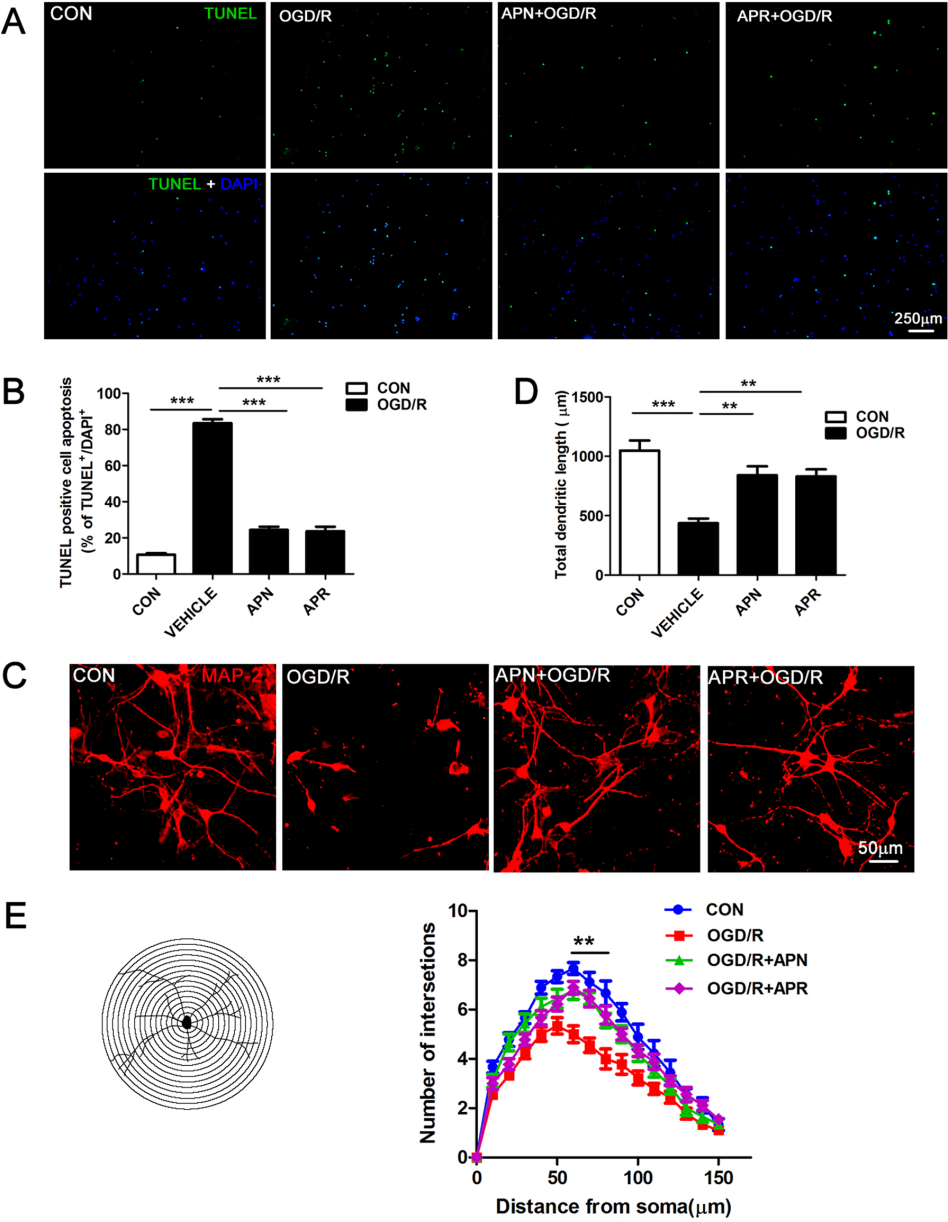
Effects of APN and APR on OGD/R-induced apoptosis and dendritic morphology in promary neurons. **(A)** Effects of APN (1 µg/ml) and APR (1 µM) on apoptotic cells stained by TUNEL in primary neurons under OGD/R. TUNEL-positive cells are green, and all cells are stained with DAPI (blue). Scale bar: 250 µm. **(B)** The bar graph indicated the effects of APN and APR on the percentage of TUNEL-positive apoptotic neurons under OGD/R (n = 4 per condition; ***P <0.001 with ANOVAs followed by Tukey’s *post hoc* test). Data are presented as mean ± SEM. **(C)** Effects of APN (1 µg/ml) and APR(1 µM) on MAP-2-positive (red) neuronal morphology under OGD/R. Scale bar: 50 µm. **(D)** The bar graph indicated the effects of APN and APR on the total dendritic length under OGD/R in primary neurons. (n = 4 per condition; **P <0.01, ***P <0.001 with ANOVAs followed by Tukey’s *post hoc* test). Data are presented as mean ± SEM. **(E)** Sholl analysis of the effects of APN and APR on the dendritic intersections under OGD/R in primary neurons (n = 9 per condition; **P <0.01 with ANOVAs followed by Tukey’s *post hoc* test). Data are presented as mean ± SEM.

It is reported that ischemic injury induces neuronal apoptosis and morphological changes of dendrite (Kanazawa et al., 2017). Thus, we investigated the roles of APN and APR on apoptosis by TUNEL. The results indicated that the percentage of TUNEL-positive apoptotic cells was 10.61 ± 0.90% in the control group, and OGD/R induced an increase of the percentage to 83.46 ± 2.16% (*P* <0.001). APR (1 µM) and APN (1 µg/ml) rescued the apoptotic cells to 23.66 ± 2.50% (*P* <0.001, **Figure 2A, B**) and 24.36 ± 1.80%, respectively (*P* <0.001, **Figure 2A, B**).

Moreover, we investigated the roles of APN and APR on morphological changes of dendrite. The MAP-2 staining results showed that the total dendritic length was 436.3 ± 39.9 µm in the OGD/R group compared with 1,048 ± 84.91 µm in the control group (*P* <0.001, **Figure 2C, D**). APN (1 µg/ml) and APR (1 µM) rescued the total dendritic length to 840.3 ± 74.83% (*P* <0.01, **Figure 2C, D**) and 830.3 ± 59.25%, respectively (*P* <0.01, **Figure 2C, D**). We further analyzed the effects of APN and APR on dendritic complexity. The Sholl analysis showed that the dendritic intersections significantly reduced between 60 µand 80 µm from the soma in OGD/R group compared with the control group (*P* <0.01, **Figure 2C, E**). Both APN and APR significantly increased the dendritic intersections between 60 and 80 µm from the soma compared with the OGD/R group (*P* <0.01, **Figure 2C, E**). Taken together, the above results suggested that APN and APR reduced the apoptosis and increased either total dendritic length and dendritic complexity on OGD/R-induced injury in primary neurons.

### Involvement of AdipoR1 in the Protective Effects of APN and APR Against OGD/R-Induced Injury

**Figure 3:**
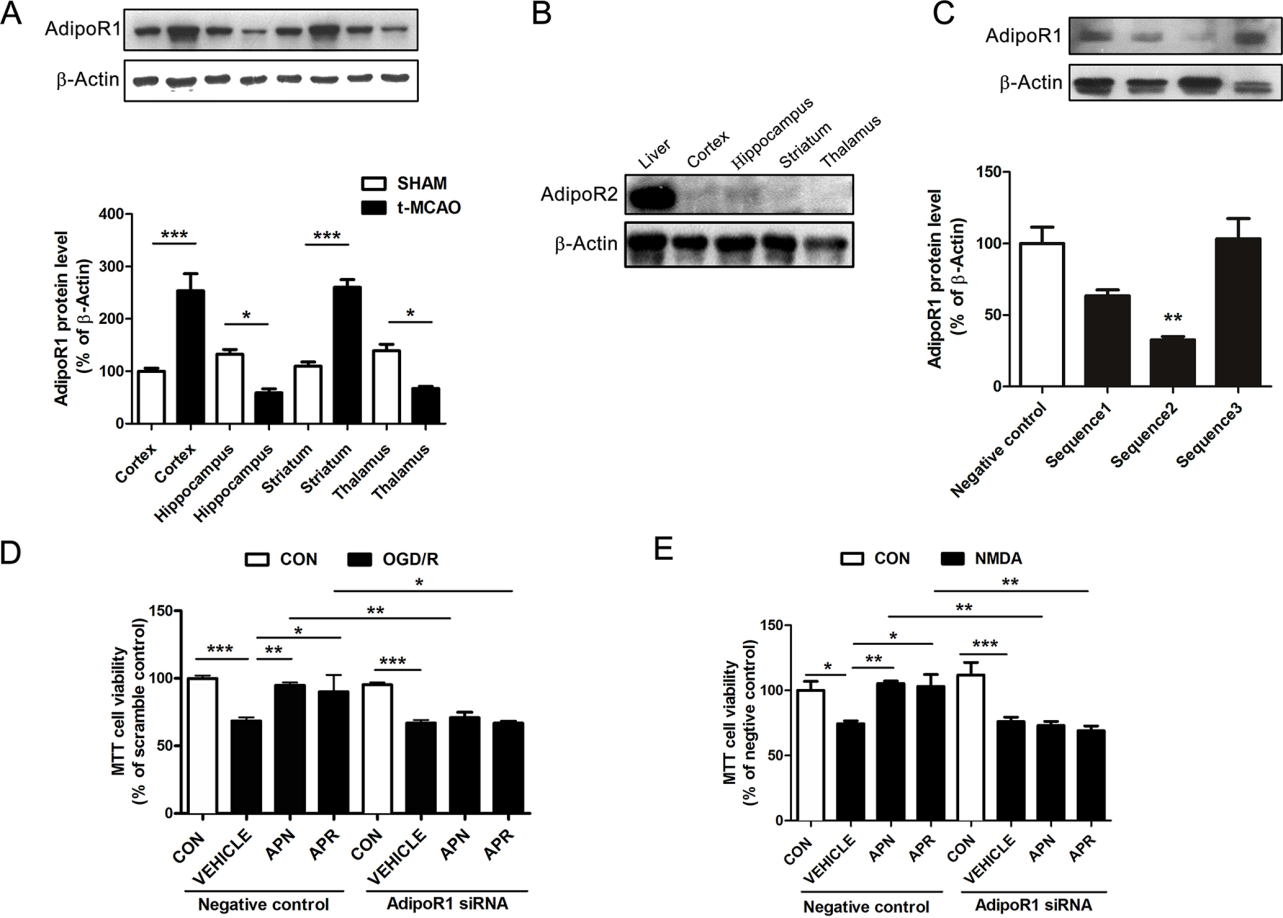
Involvement of AdipoR1 in the protective effects of APN and APR against OGD/R-induced injury in primary neurons. **(A)** Representative Western blots and the bar graph showing the AdipoR1 expression in different brain regions under tMCAO-induced cerebral injury (n = 3 per condition; *P <0.05, ***P <0.001 with ANOVAs followed by Tukey’s *post hoc* test). Data are presented as mean ± SEM. **(B)** Representative Western blots showing the AdipoR2 expression in the liver and brain. **(C)** Representative Western blots and the bar graph showing the AdipoR1 expression interfered by three different siRNA sequences cultured cortical neurons (n = 3 per condition; **P <0.01 vs. negative control group with ANOVAs followed by Tukey’s *post hoc* test). Data are presented as mean ± SEM. **(D)** The cell viability tested by MTT assay showing the effect of AdipoR1 siRNA on the protections of APN and APR against OGD/R in primary neurons (n = 8–9 per condition; *P <0.05, **P <0.01, ***P <0.001 with ANOVAs followed by Tukey’s *post hoc* test). Data are presented as mean ± SEM. **(E)** The cell viability tested by MTT assay showing the effect of AdipoR1 siRNA on the protection of APN and APR against NMDA in primary neurons (n = 9 per condition; *P <0.05, **P <0.01, ***P <0.001 with ANOVAs followed by Tukey’s *post hoc* test). Data are presented as mean ± SEM.

Reports show that AdipoR1 is widely expressed in the brain, whereas AdipoR2 is barely expressed in the brain (Hug et al., 2004). To investigate the role of APN receptors in ischemia, we examined their expression by Western blot. Results indicated that AdipoR1 was extensively expressed in the brain cortex, hippocampus, striatum, and thalamus (**Figure 3A**). Interestingly, the expression of AdipoR1 increased dramatically in the cortex and striatum, regions that are highly vulnerable to ischemia (Hammond et al., 1994; Miladinovic et al., 2015) (**Figure 3A**). This might be an endogenous protection against ischemic injury and implicated an important role of AdipoR1 in ischemia. We also examined the expression of AdipoR2 in the brain. Results showed that AdipoR2 is highly expressed in the liver, but barely expressed in brain regions including cortex, hippocampus, striatum, and thalamus (**Figure 3B**). These results reminded us that AdipoR1 but not AdipoR2 might play an important role in the CNS.

To further investigate the role of AdipoR1 in ischemia, AdipoR1 siRNA was transfected in primary neurons. Three siRNA fragments were applied, and the results showed that the effective siRNA fragment is sequence 2 (reduced to 32.71± 2.28% of negative control, *P* <0.01, **Figure 3C**). Thus, sequence 2 was selected for the later study. The results indicated that the protective effects of APN against OGD/R was reversed completely by AdipoR1 siRNA (from 94.94 ± 1.96% to 70.88 ± 4.04%, *P* <0.01, **Figure 3D**). In addition, the protection of APR against OGD/R was also reversed by AdipoR1 silencing (from 90.03 ± 12.48% to 66.93 ± 1.41%, *P* <0.05,**** **Figure 3D**). Moreover, we also observed similar effects of APN (from 105.3 ± 1.86% to 73.03 ± 3.18%, *P* <0.01, **Figure 3E**) and APR (from 103.1 ± 9.14% to 69.07 ± 3.62%, *P* <0.01, **Figure 3E**) in NMDA-induced excitotoxicity.

### Involvement of AMPK Pathway in the Protective Effects of APN and APR on OGD/R-Induced Injury

**Figure 4:**
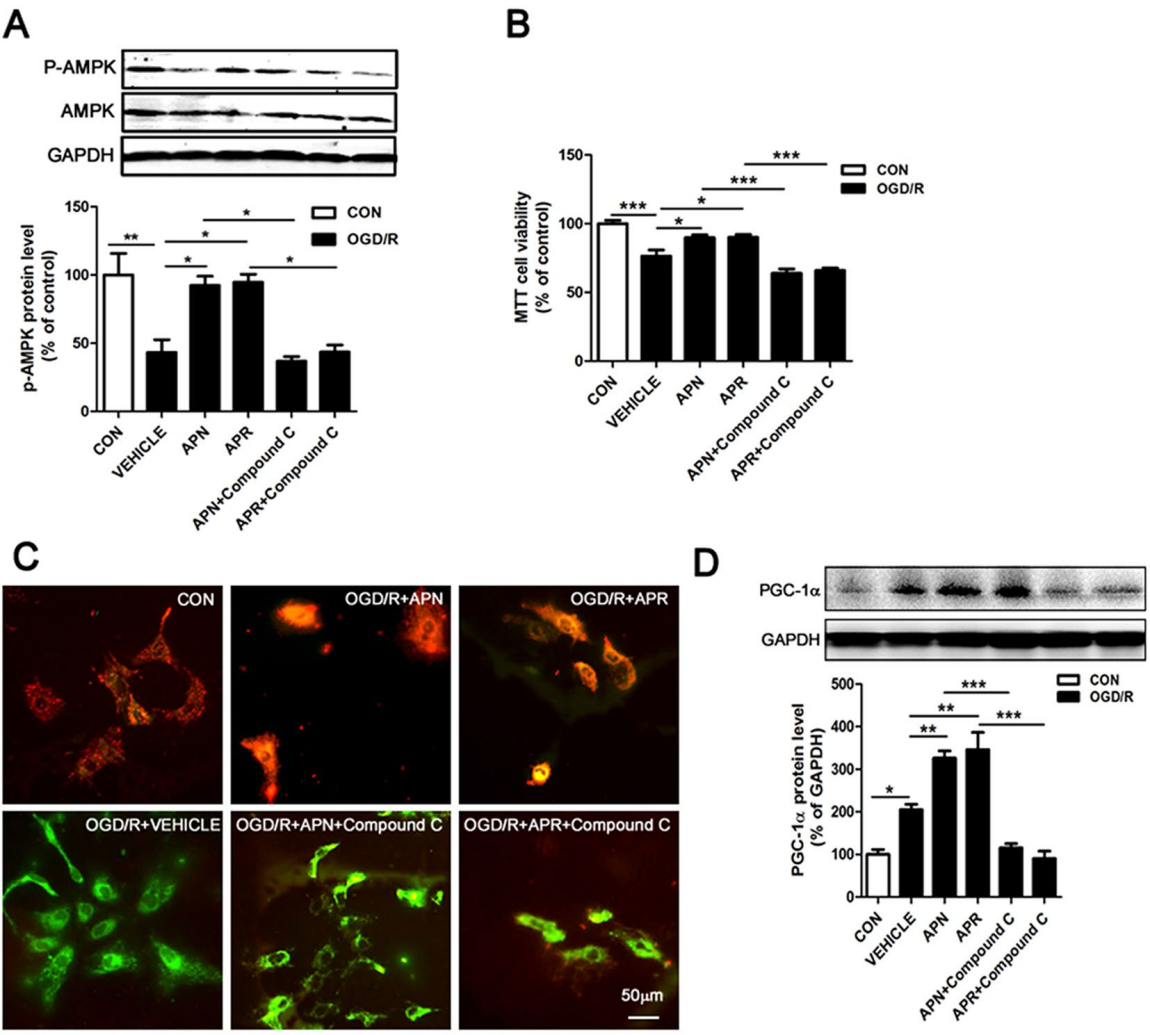
Involvement of AMPK pathway in the protective effects of APN and APR against OGD/R-induced injury. **(A)** Representative Western blots and bar graph showing the effects of APN (1 µg/ml, 0.5 h before OGD), APR (1 µM, 0.5 h before OGD) and compound C (10 µM, 1 h before OGD) on AMPK phosphorylation under OGD/R in primary neurons (n = 3 per condition; *P <0.05, **P <0.01 with ANOVAs followed by Tukey’s *post hoc* test). Data are presented as mean ± SEM. **(B)** Effects of compound C (10 µM, 1 h before OGD) on the protections of APN (1 µg/ml, 0.5 h before OGD) and APR (1 µM, 0.5 h before OGD) against OGD/R-induced injury in primary neurons by MTT assay (n = 8 per condition; *P <0.05, ***P <0.001 with ANOVAs followed by Tukey’s *post hoc* test). Data are presented as mean ± SEM. **(C)** Representative JC-1 fluorescence images showing the effects of APN (1 µg/ml, 0.5 h before OGD) and APR (1 µM, 0.5 h before OGD) on OGD/R-induced injury in primary neurons. Red fluorescence indicates a polarized state and green fluorescence indicates a depolarized state. Scale bar: 50 µm. **(D)** Representative Western blots and the bar graph showing the PGC-1α expression treated by compound C (10 µM, 1 h before OGD), APN (1 µg/ml, 0.5 h before OGD), and APR (1 µM, 0.5 h before OGD) in primary neurons under OGD/R-induced injury (n = 4 per condition; *P <0.05, **P <0.01,***P <0.001 with ANOVAs followed by Tukey’s post *hoc test*). Data are presented as mean ± SEM.

Studies show that APN binds with AdipoR1 to activate AMPK pathway (Thundyil et al., 2012). APN improves numerous neurological diseases through AMPK signaling (Chan et al., 2012; Yan et al., 2013; Xu et al., 2018). To investigate the signaling pathway of the protective effects offered by APN and APR, we analyzed the AMPK phosphorylation. The Western blot results showed that the expressions of p-AMPK was significantly lower in the OGD/R group in primary neurons compared with the control group (reduced to 43.16 ± 9.36% of control, *P* <0.01, **Figure 4A**), suggesting OGD/R inactivated AMPK pathway. However, the expression of p-AMPK was significantly higher in APN group (increased to 92.27 ± 6.78% of control, *P* <0.05, **Figure 4A**) and APR group (increased to 94.63 ± 5.90% of control, *P* <0.05, **Figure 4A**) compared with the vehicle group, suggesting APN and APR promoted the activation AMPK pathway. Moreover, compound C, an inhibitor of p-AMPK, reversed the p-AMPK level offered by both APN (reduced to 36.87 ± 3.34% of control, *P* <0.05, **Figure 4A**) and APR (reduced to 43.55 ± 5.11% of control, *P* <0.05, **Figure 4A**), suggesting the effectiveness of compound C.

To further investigate the involvement of AMPK in the protections of APN and APR on OGD/R-induced injury, the cell viability was assessed when the inhibitor of p-AMPK (compound C) was administrated. The results showed that the cell viability markedly decreased both in the APN+ compound C group compared with the APN group (from 89.78 ± 2.09% to 63.99 ± 3.02% of control, *P* <0.001, **Figure 4B**) and in the APR+ compound C group compared with the APR group (from 90.18 ± 2.01% to 65.97 ± 1.91% of control, *P* <0.001, **Figure 4B**), suggesting an important role of AMPK pathway in the protective effects of APN and APR on OGD/R-induced injury in primary neurons. Results above revealed that the protective effects of APN and APR on OGD/R-induced injury might be at least in part via AMPK pathway.

### Effects of APN and APR on Mitochondrial Dysfunction on OGD/R-Induced Injury

It is reported that impaired mitochondrial function occurred in ischemia (Galluzzi et al., 2009; Yang et al., 2018), and AMPK plays an important role in the maintenance of mitochondrial function (Rabinovitch et al., 2017). To reveal the effects of APN and APR on mitochondrial function, we examined the ΔΨm on OGD/R-induced injury in primary neurons. The results indicated that OGD/R induced a significant dissipation of the ΔΨm (**Figure 4C**); however, both APN and APR reversed the dissipation of the ΔΨm on OGD/R-induced injury (**Figure 4C**). Moreover, to investigate the role of AMPK in the maintenance of mitochondrial function offered by APN and APR, the inhibitor of p-AMPK (compound C) was administrated. The results showed that compound C completely reversed the ΔΨm in the APN+ compound C group compared with the APN group, and similar results was observed in the APR+ compound C group compared with the APR group (**Figure 4C**). Above all, these results showed that APN and APR maintained the mitochondrial function through AMPK pathway.

### Effects of APN and APR on the Expression of PGC-1α

Studies show that decreased levels of APN causes mitochondrial dysfunction by decreasing the PGC-1α expression in diabetic mice (Iwabu et al., 2010; Yan et al., 2013). To investigate whether APN and APR protect the mitochondrial function through up-regulation of PGC-1α, we assessed the expression of PGC-1α by Western blot. The results indicated that the PGC-1α expression was significantly increased in both APN group (326.2 ± 16.93% of control, *P* <0.01, **Figure 4D**) and APR group (346.2 ± 41.32% of control, *P* <0.01, **Figure 4D**) compared with the vehicle group (204.8 ± 12.84% of control, *P* <0.05, **Figure 4D**). Moreover, to study the role of AMPK in the up-regulation of PGC-1α offered by APN and APR, we inhibited the p-AMPK by compound C. The results showed that the PGC-1α protein level decreased significantly in the APN+ compound C group (115.3 ± 9.99% of control, *P* <0.001, **Figure 4D**) compared with the APN group and in the APN+ compound C group (90.12 ± 17.63% of control, *P* <0.001, **Figure 4D**) compared with the APR group. Taken together, these results indicated that with OGD/R-induced injury, APN and APR up-regulated PGC-1α level is through AMPK pathway.

### APN and APR Alleviates the Cerebral Ischemia/Reperfusion (I/R)-Induced Injury Through AMPK Pathway *In Vivo*

**Figure 5:**
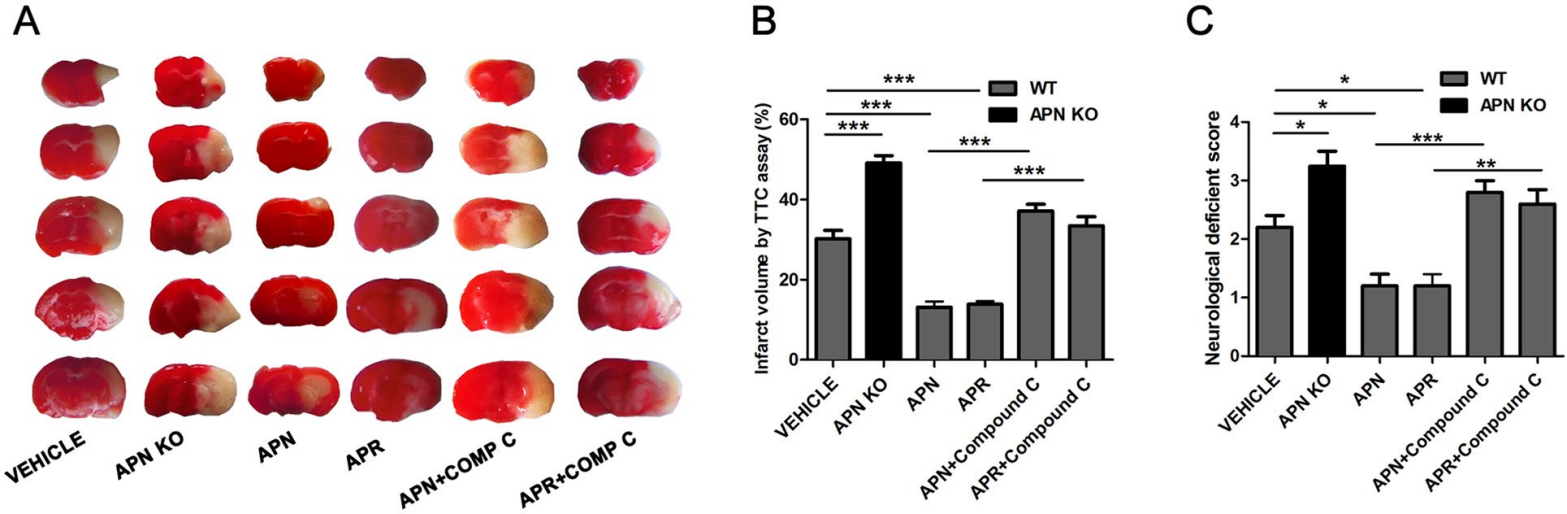
APN and APR alleviate the tMCAO-induced injury through AMPK pathway. **(A)** Brain sections stained by 2,3,5-Triphenyltetrazolium chloride (TTC) from WT and APN-/- mice showing the infarct area in those receiving saline, APN (*i.c.v.*, 0.3 µg/mouse, 0.5 h before tMCAO), APR (i.c.v., 1 µg/mouse, 0.5 h before tMCAO), and compound C (*i.c.v.*, 40 µg/mouse, 1 h before tMCAO). The bar graph showing the infarct volume **(B)** and neurological scores **(C)** (n = 4–5 per condition; *P <0.05, **P <0.01, ***P <0.001 with ANOVAs followed by Tukey’s *post hoc* test). Data are presented as mean ± SEM.

As it was reported that APN is protective against cerebral I/R-induced injury in diabetic mice in a AdipoR1-dependent way (Song et al., 2015). However, the role of APN and APR on I/R-induced injury in mice remain to be clarified. To investigate the role of APN in cerebral I/R injury, the APN knockout (APN-KO) mice was used. The results indicated that the infarct volume increased significantly in the APN-KO group (49.18 ± 1.81%, *P* <0.001, **Figure 5A, B**) compared with the vehicle group (30.25 ± 2.07%, **Figure 5A, B**). The infarct volume increased significantly in the APN-KO group (49.18 ± 1.81%, *P* <0.001, **Figure 5A, B**) compared with the vehicle group (30.25 ± 2.07%, **Figure 5A, B**). The neurological deficit scores increased in the APN-KO group (3.25 ± 0.25, *P* <0.05, **Figure 5C**) compared with the vehicle group (2.20 ± 0.2, **Figure 5C**). To further study the role of APN and APR in ischemia, we assessed the effects of APN and APR on I/R-induced injury. The I/R-induced infarct volume reduced to 13.13 ± 1.43% in the APN group and 13.86 ± 0.83% in the APR group compared with the vehicle group (*P* <0.001, **Figure 5A, B**). The I/R-induced neurological deficient scores reduced to 1.20 ± 0.20 in the APN group and 1.17 ± 0.17 in the APR group compared with the vehicle group (*P* <0.05, **Figure 5C**). Moreover, to investigate the role of AMPK in the protective effects of APN and APR, compound C was administrated. The infarct volume increased significantly in both the APN+ compound C group compared with the APN group. (37.18 ± 1.63%, *P* <0.001, **Figure 5A, B**) and the APR+ compound C group (33.45 ± 2.26%, *P* <0.001, **Figure 5A, B**) compared with the APR group. The neurological deficit scores increased in both the APN+ compound C group (2.8 ± 0.2, *P* <0.01, **Figure 5C**) compared with the APN group and the APR+ compound C group (2.60 ± 0.24, *P* <0.001, **Figure 5C**) compared with the APR group. Above all, these results showed that APN and APR alleviated the tMCAO-induced injury no matter on infarct volume or neurological deficiency through AMPK pathway.

## Discussion

APN has been known to regard to glucose regulation and fatty acid oxidation (Diez and Iglesias, 2003). APN binds with its receptors (AdipoR1 and AdipoR2) to regulate cell functions (Scherer et al., 1995; Mao et al., 2006; Thundyil et al., 2012). Studies show that APN protects against glutamate-induced excitotoxicity in HT22 hippocampal neurons (Wang et al., 2018; Yue et al., 2016). APN also protects against ischemia in diabetic mice (Song et al., 2015). It is known that cortex and striatum are highly vulnerable to ischemia (Hammond et al., 1994; Miladinovic et al., 2015); thus, we investigated that role of APN and APR on primary cortical neurons. In this study, our findings suggested that APN and APR were all protective against OGD/R-induced injury in primary cortical neurons. In addition, we found that APN and APR were also protective against tMCAO-induced ischemic injury in adult mice, and APN-KO aggravated the tMCAO-induced injury. Therefore, our findings suggested that APN might protect neurons against OGD/R-induced injury *in vitro* and tMCAO-induced injury *in vivo*.

It had been reported that AdipoR1 is widely expressed in the brain, whereas AdipoR2 is barely expressed in the brain (Hug et al., 2004), and AdipoR1 is important for neuronal survival under pathological conditions (Thundyil et al., 2012). We also found that AdipoR1 level increased in cortex and striatum, a region that is vulnerable in ischemia, suggesting it might be an endogenous protective mechanism through AdipoR1. Moreover, the finding of the study also showed that AdipoR2 is highly expressed in the liver, but barely expressed in brain regions including cortex, hippocampus, striatum, and thalamus. Osmotin, as an APN homolog, protects H9c2 cells against I/R through AdipoR1-dependent PI3K/AKT signaling (Liu et al., 2017). APN attenuates neuronal apoptosis induced by hypoxia-ischemia via AdipoR1/APPL1 pathway in neonatal rats (Xu et al., 2018). Based on these results, we investigated the role of AdipoR1 in OGD/R-induced injury in primary neurons. This study showed that the protection of APN and APR against OGD/R- and NMDA-induced injury were compromised by siRNA of AdipoR1, suggesting that the protective effects were AdipoR1-dependent.

AMPK is a major downstream component of APN signaling that acts as the cellular energy sensor (Thundyil et al., 2012). Studies have shown that metabolic effects mediated by APN in peripheral tissues such as skeletal muscle and liver through activation of AMPK (Yamauchi et al., 2002). APN is protective against Parkinson’s disease (PD) through AMPK activating (Li et al., 2014). AMPK activation is also involved in APN mediated protection against cardiovascular diseases (Kobayashi et al., 2004). APN protects neurons against hypoxia-ischemia-induced injury via AMPK pathway *in vivo* (Xu et al., 2018). However, further studies are required to determine whether APN and APR protect against ischemic injury through AMPK pathway. As expected, this study showed that compound C, an AMPK activity inhibitor, reversed the protective effects offered by APN and APR on OGD/R-induced and NMDA-induced injury *in vitro*, and tMCAO-induced injury *in vivo*. Above all, the study showed that the abovementioned results revealed that the protective effects of APN and APR on I/R-induced injury might be at least in part via AMPK pathway.

Two early hallmarks of neuronal excitotoxicity are mitochondrial dysfunction and the formation of focal swellings along the length of the dendrites (Greenwood and Connolly, 2007). APN deficiency induces mitochondrial dysfunction and oxidative stress (Lin et al., 2014). Lacking AdipoR1 induces myocardial mitochondrial dysfunction (Koentges et al., 2015), and AMPK is a critical regulator involved in initiating mitochondrial biogenesis (Zong et al., 2002). These reports indicated that APN and downstream signaling AdipoR1/AMPK is beneficial to mitochondrial dysfunction. APN treatment activates AMPK and PGC-1α, increases mitochondrial biogenesis, and attenuates mitochondrial disorders in the heart (Yan et al., 2013). APN also induces PGC-1α up-regulation and increased mitochondria content in myocytes through AdipoR1/AMPK (Iwabu et al., 2010). Based on the abovementioned studies, we investigated the effects of APN and APR on mitochondrial function and PGC-1α expression on OGD/R-induced injury in primary neurons. This study indicated that APN and APR maintained the mitochondrial function through AMPK pathway. Moreover, APN and APR also up-regulated the PGC-1α level through AMPK pathway. Taken together, our results showed that APN and APR might enhance the PGC-1α level and repair the mitochondrial dysfunction through AMPK signaling.

## Conclusion

The finding of this study showed that APN and APR were all protective against OGD/R-induced injury in primary neurons *in vitro*. This protective effect was mainly related to anti-apoptosis properties, increased either total dendritic length or dendritic complexity and repaired mitochondrial dysfunction. In addition, AdipoR1 and the downstream AMPK/PGC-1α signaling were related to the protection against OGD/R-induced injury. Moreover, APN and APR alleviated the tMCAO-induced injury on infarct volume and neurological deficiency through AMPK pathway *in vivo*.

## Ethics Statement

This study was carried out in accordance with the recommendations of the Animal Experimentation Committee of Binzhou Medical University Hospital. The protocol was approved by the Animal Experimentation Committee of Binzhou Medical University Hospital.

## Author Contributions

HY designed the experiments and analyzed the data. HY and BL wrote the article. HY, BL, JL, JW, FS, SJ, FH, DW, DL, and CL performed the experiments.

## Funding

Work of the authors was supported by grants from the National Natural Science Foundation of China (81500930), the Natural Science Foundation of Shandong Province (ZR2014HQ014 and ZR2017BC047), and University Science and Technology Plan of Shandong Province (J13LL06).

## Conflict of Interest Statement

The authors declare that the research was conducted in the absence of any commercial or financial relationships that could be construed as a potential conflict of interest.
